# Internet-mediated physiotherapy and pain coping skills training for people with persistent knee pain (IMPACT – knee pain): a randomised controlled trial protocol

**DOI:** 10.1186/1471-2474-15-279

**Published:** 2014-08-13

**Authors:** Fiona Dobson, Rana S Hinman, Simon French, Christine Rini, Francis Keefe, Rachel Nelligan, J Haxby Abbott, Christina Bryant, Margaret P Staples, Andrew Dalwood, Kim L Bennell

**Affiliations:** Centre for Health, Exercise and Sports Medicine, Department of Physiotherapy, School of Health Sciences, The University of Melbourne, Alan Gilbert Building 161 Barry St, Carlton, Vic 3053 Australia; School of Rehabilitation Therapy, Faculty of Health Sciences, Queen’s University, Kingston, Ontario Canada; Gillings School of Global Public Health, Department of Health Behavior, University of North Carolina, Chapel Hill, Raleigh, NC USA; Department of Psychiatry and Behavioral Sciences, Duke University, Durham, NC USA; Centre for Musculoskeletal Outcomes Research, Department of Surgical Sciences, Dunedin School of Medicine, University of Otago, Dunedin, New Zealand; Psychological Sciences, University of Melbourne and Royal Women’s Hospital, Melbourne, Vic Australia; Department of Epidemiology at Cabrini, Cabrini Institute and Monash University, Malvern Victoria, USA; Physioworks Health Group, Camberwell, Victoria Australia

**Keywords:** Knee osteoarthritis, Knee pain, Physiotherapy, Pain coping skills, Internet, Health care delivery, Randomised control trial

## Abstract

**Background:**

Persistent knee pain in people over 50 years of age is often attributable to knee osteoarthritis (OA), a common joint condition that causes physical and psychological dysfunction. Exercise and pain coping skills training (PCST) can help reduce the impact of persistent knee pain, however, access to health professionals who deliver these services can be challenging. With increasing access to the Internet, remotely delivered Internet-based treatment approaches may provide alternatives for healthcare delivery. This pragmatic randomised controlled trial will investigate whether an Internet-delivered intervention that combines PCST and physiotherapist-guided exercise (PCST + Ex) is more effective than online educational material (educational control) in people with persistent knee pain.

**Methods/Design:**

We will recruit 148 people over 50 years of age with self-reported persistent knee pain consistent with knee OA from the Australian community. Following completion of baseline questionnaires, participants will be randomly allocated to access a 3-month intervention of either (i) online educational material, or (ii) the same online material plus an 8-module (once per week) Internet-based PCST program and seven Internet-delivered physiotherapy sessions with a home exercise programs to be performed 3 times per week. Outcomes will be measured at baseline, 3 months and 9 months with the primary time point at 3 months. Primary outcomes are average knee pain on walking (11-point numeric rating scale) and self-reported physical function (Western Ontario and McMaster Universities Osteoarthritis Index subscale). Secondary outcomes include additional measures of knee pain, health-related quality-of-life, perceived global change in symptoms, and potential moderators and mediators of outcomes including self-efficacy for pain management and function, pain coping attempts and pain catastrophising. Other measures of adherence, adverse events, harms, use of health services/co-interventions, and process measures including appropriateness and satisfaction of the intervention, will be collected at 3, 6 and 9 months.

**Discussion:**

The findings will help determine the effectiveness and acceptability of Internet access to a combination of interventions that are known to be beneficial to people with persistent knee pain. This study has the potential to guide clinical practice towards innovative modes of healthcare provision.

**Trial registration:**

Australian New Zealand Clinical Trials Registry reference: ACTRN12614000243617.

**Electronic supplementary material:**

The online version of this article (doi:10.1186/1471-2474-15-279) contains supplementary material, which is available to authorized users.

## Background

Osteoarthritis (OA) is the leading cause of persistent knee pain in people over 50 years old [1]. In the Global Burden of Disease 2010 study [2], knee OA, together with hip OA, was ranked as the eleventh highest contributor to global disability with a global age-standardised prevalence of knee OA estimated at 3.8% [3]. At the individual level, persistent pain due to knee OA can be debilitating. It leads to loss of function, reduced quality of life [4], and psychological disability [5]. Many people with knee OA experience co-morbidities such as obesity, depression and cardiovascular disease that add further burden to the disease. With an aging and increasingly obese world population, that are projected to increase the prevalence of knee OA pain, there is an urgent need for accessible and effective treatments that improve patient symptoms and function, while minimising the costs to both patients and society.

Interventions that foster self-management, such as exercise, are considered fundamental to managing knee pain due to knee OA. Considerable evidence supports the use of strengthening exercises to reduce pain and improve function in this patient population [6–10]. Exercise is universally recommended by clinical guidelines [11] regardless of age, disease severity, pain intensity, functional levels and co-morbidities. Meta-analyses [12, 13] consistently report benefits of all types of exercise compared to education or no treatment and exercise programs that combine strengthening, flexibility and aerobic exercise seem to provide the best benefits for improving pain and function [14]. Importantly, exercise is regarded as a safe intervention in patients with knee OA with few contraindications or adverse events [15].

Although regular exercise can reduce physical impairments and improve participation in everyday life activities [16], meta-analysis has consistently revealed it produces relatively modest improvements in pain and function [13, 14, 17]. Furthermore, some patients experience increased pain when they start exercising leading them to discontinue this treatment. Exercise primarily targets biomedical factors that can influence pain and does not directly address psychological factors such as self-efficacy for pain control, adaptive pain coping skills and overly negative thinking about pain (pain catastrophising). As low self-efficacy and ineffective pain coping strategies are common in chronic pain populations [5, 18, 19], interventions that target these key psychological factors in addition to biomedical factors may provide more powerful benefit. Accordingly and consistent with a biopsychosocial approach to chronic pain management, there has been growing interest in interventions that combine exercise and psychological treatments.

Cognitive behavioural therapy is the most frequently studied psychological intervention for pain control in individuals with arthritis [20]. Growing evidence supports the use of pain coping skills training (PCST), an approach based on cognitive behavioural principles, to improve pain and psychological functioning in chronic pain conditions [21–23]. More recent evidence shows that PCST combined with exercise, delivered by specially trained physiotherapists, is effective in people with knee pain due to knee OA [24]. Despite the advantages of using an intervention that combines exercise and PCST, access to specialised health professionals to deliver this combined intervention is not readily available. As PCST is currently in the domain of specialised psychologists, people with OA knee pain are not always able to access specialised health professionals to deliver this combined intervention, especially if they live in rural and remote areas [25, 26].

The Internet has been increasingly used as a time efficient and convenient method to deliver health interventions [27]. It is estimated that around 34% of the world’s population (roughly 2.4 billion people) use the Internet [28]. Its use is growing in all segments of our society, including within the home, where there has been a 566% increase in worldwide use over the last decade [28]. Interestingly, older adults are the age group with the fastest growing Internet usage [29]. Therefore, the Internet represents a viable delivery mode to allow interventions to reach a large number of individuals, including older adults living in remote areas.

Members of our research team (CR, FK) recently developed an automated, Internet-based PCST program called PainCOACH to improve access to PCST for people with persistent OA pain. This program is likely to be more cost effective than face-to-face PCST because it eliminates the need for trained professionals to lead weekly face-to-face sessions [30–33]. It also has the potential to be more convenient than face-to-face PCST because people with OA can use it at home at a time that suits them. Similarly, teleconferencing and Internet-based telerehabilitation have been used as alternative methods for delivering physiotherapy and exercise [34]. These methods have been found to be useful for people with persistent knee pain [35] and are comparable to conventional face-to-face physiotherapy for people following knee joint replacements [36, 37]. Furthermore, both health professionals and patients using Internet-based methods reported levels of satisfaction that were no different than those reported by health professionals and patients using traditional consultation methods [38]. However, the combination of delivering both PCST and physiotherapy over the Internet has not yet been rigorously evaluated in people with persistent knee pain consistent with knee OA.

The primary objective of this pragmatic randomised controlled trial (RCT) is to evaluate whether an Internet-based intervention that combines PCST and physiotherapist-guided exercise (PCST + Ex), is more effective than online educational material (educational control) in people with persistent OA knee pain. We hypothesise that PCST + Ex will be more effective in reducing pain and improving self-reported physical function after 3 months than the educational control intervention. Secondary hypotheses are (i) the PCST + Ex will be more effective in reducing pain and improving self-reported physical function after 9 months than the educational control intervention; and (ii) there will be greater improvements in health-related quality of life and psychological outcomes, as well as a better participant-perceived response to treatment in the PCST + Ex after 3 months and 9 months than the education control intervention.

## Methods/Design

### Trial design

**Figure 1:**
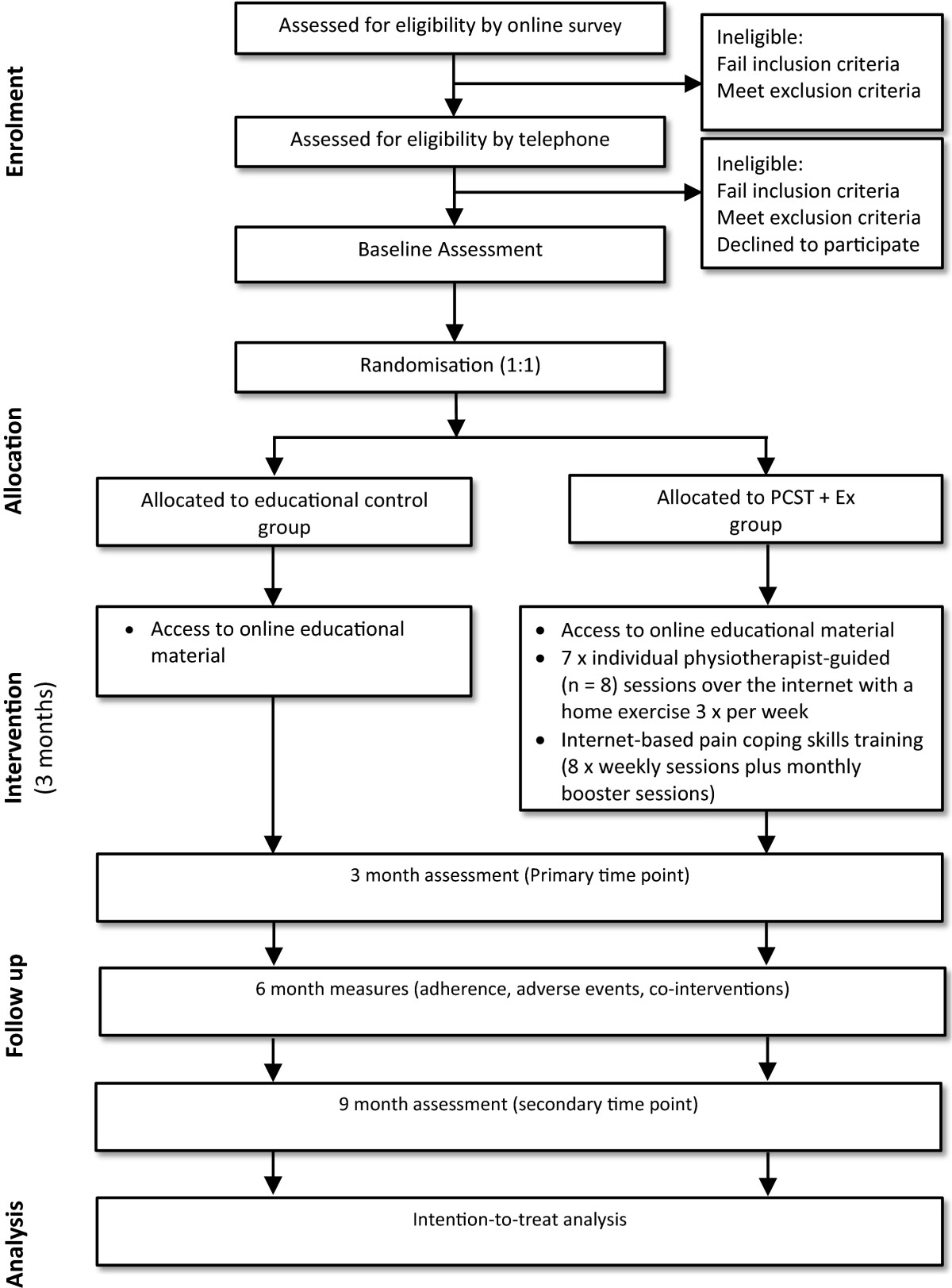
**Flow diagram of study protocol.**

Parallel-design 2-arm pragmatic RCT, with a 3-month intervention and outcomes assessed at 3 and 9 months from baseline. The primary outcome time point will be 3 months. Reporting of the trial will comply with the CONSORT statement for randomised trials and the extension for trials assessing non-pharmacologic treatments [39, 40] (Figure 1).

### Participants

We will recruit 148 participants aged ≥ 50 years with persistent OA knee pain from the Australian community via advertisements, media campaigns, social media (e.g. Facebook and Twitter), university websites, our research volunteer database, and other external databases of volunteers who have registered as being interested in participating in clinical research. Participants will be included if they i) are aged ≥ 50 years; ii) report persistent knee pain for more than 3 months and for most days of the past month; iii) report a minimum average knee pain intensity during walking of 4 on an 11-point numerical rating scale (NRS, with terminal descriptors of ‘no pain’ and ‘worst pain possible’) in the past week; iv) report at least mild-moderate physical dysfunction (score > 20 on the Western Ontario McMaster Universities (WOMAC) physical function subscale); and v) have an active email account and access to a computer with a broadband Internet connection.

Participants will not be eligible if they: i) have had a knee joint replacement on the painful knee side; ii) are on the waiting list for joint replacement surgery; iii) have undergone intra-articular corticosteroid injection or knee surgery to either knee within past 6 months or are planning joint surgery in the next 9 months; iv) are currently receiving or have received treatment for knee pain (e.g. physiotherapy, chiropractic, osteopathy, acupuncture or psychologist)in the last six months from a health practitioner, or if they have participated in a muscle strengthening exercise program or a pain coping skills program in the last 6 months; v) have a systemic arthritic condition; vi) have any neurological conditions that affect the lower-limb and limit their ability to exercise safely (such as a stroke, multiple sclerosis, polio, neuropathy, peripheral nerve disease or Parkinson’s disease); vii) have any other major joint pain (e.g. back, hip or ankle) to a greater extent than their knee pain that currently limits the ability to exercise; viii) have self-reported high-level depression (score of >21 on the depression subscale of the Depression, Anxiety and Stress Scale) and/or; ix) are not fluent in written and spoken English.

### Procedure

Figure 1 outlines the trial phases. Eligibility of volunteers will be initially confirmed via an online screening survey followed by a telephone interview. Baseline, 3-month (primary time point) and 9-month (secondary time point) assessments will be conducted via the Internet using online assessment surveys. Additionally, participants will complete log books to capture adherence and adverse event data. These will be either transcribed by participants to an online survey or mailed back to researchers, as preferred by each participant. Ethical approval has been obtained from the University of Melbourne Human Research Ethics Committee (No. 1339459). All participants will provide written informed consent. For participants with bilaterally eligible knees, only the most painful knee (as identified by the participant) will be evaluated.

### Randomisation and allocation concealment

On completion of baseline assessment, participants will be consecutively randomised to one of two groups: 1) educational control (online educational material) or 2) intervention group (online educational material, Internet-based PCST and physiotherapy-guided exercise program delivered via the Internet). Computer generated randomisation will be conducted by random permuted blocks of size 2-8 stratified by gender (male or female) and residency (metropolitan or rural), prepared by our study biostatistician (MS). To conceal randomization, consecutively numbered, sealed, opaque envelopes will be prepared by a researcher with no other involvement in the study. The envelopes will be stored in a locked location and will be opened in sequence within each stratum to reveal group allocation.

All primary and secondary outcome data will be completed online and thus will be automatically entered into a database. By necessity, participants and physiotherapists will be unblinded to group allocation; however, participants will remain blinded to research hypotheses. The statistician will be blinded to group allocation until completion of the statistical analyses.

### Interventions

#### Education control condition

Participants allocated to the educational control group will receive a specific URL-link to access online educational material about knee OA. This educational material comprises information sheets produced and provided by Arthritis Australia that is also available to the general public via their website (http://www.arthritisaustralia.com.au). It will contain a recommended reading list that covers topics such as exercise and physical activity, managing pain, and general information about knee pain and knee OA. It will also contain an additional reading list that will cover topics such as emotions, healthy eating, and complementary therapies and medications. Participants in the control group are encouraged to access the education material at their own leisure and pace.

#### PCST + Ex condition

Participants in the PCST + Ex group will receive three inter-related interventions that are delivered via the Internet. First, they will receive the same specific URL-link to access the same online educational material as the educational control group. Second, they will receive access to PainCOACH, which includes eight 35- to 45-minute modules that each teach a pain coping skill shown to help people manage persistent OA pain. The modules are designed to be completed at the rate of one per week. Participants will also receive seven physiotherapy consultations delivered over the Internet using a video telephony service, Skype™. The initial session will be 45-minutes, followed by 30-minute sessions thereafter. During these consultations, participants will be prescribed an individualised home-based exercise program to be performed three times weekly. The weekly PainCOACH sessions will occur during the 3-month intervention period in Weeks 1-8, with a booster session at Week 11. The physiotherapy sessions will occur in Weeks 2, 3, 4, 6, 8, 10 and 12 of the 3-month intervention period. Participants will receive further monthly PainCOACH boosters during the 6 months following the intervention period, where they will be encouraged to review the final module of the program as well as revisit useful/meaningful modules. They will also receive monthly email reminders to encourage them to continue their exercises at home and practice their pain coping skills.

Prior to beginning the intervention, participants will receive Skype set-up training, including instructions on how to set up their web-camera and their exercise environment. They will also be given an instructional manual and password access to PainCOACH from the researchers. Any equipment required for the exercises (e.g. elastic bands, ankle weights, web camera if required, and pedometer if required) will be mailed to participants before the intervention commences.

*The PCST component* will be delivered using the PainCOACH program, which translates key therapeutic components of face-to-face PCST [41] for delivery in eight highly interactive, automated training sessions (i.e., it does not require interaction with a therapist). The program’s eight modules provide interactive training in a cognitive or behavioural pain coping skill. Module 1 provides an overview describing the PainCOACH program, PCST, and a therapeutic rationale to explain how pain coping skills can help people manage OA pain [42]. This overview is followed by training in the first pain coping skill: progressive muscle relaxation. Modules 2-7 teach brief relaxation skills, activity-rest cycling, pleasant activity scheduling, cognitive restructuring, pleasant imagery, and problem solving. Module 8 consolidates learning and teaches strategies for long term skill use. A summary of the content and flow of PainCOACH modules is provided in Table 1. Consistent with a face-to-face PCST protocol, participants are asked to practice each new skill they learn. For each new skill, participants’ completion of practices and experiences with them are reviewed at the beginning of the subsequent week’s module.

**Table 1:** **Summary of the internet-based PainCOACH content**

Module number	Coping skill	Content
**1**	**Progressive relaxation**	Teach Gate Control Theory (how thoughts, feelings, and actions affect and are affected by pain). Introduce and demonstrate progressive relaxation with animation; walk user through use of the technique and active practice; help user identify/address circumstances that might impede relaxation and chose strategies to overcome obstacles; plan regular practice times; set practice goal.
**2**	**Mini-practices**	Review prior session content and practices; Introduce and demonstrate “mini-practices” (brief relaxation) with animation; walk user through use of the technique and active practice, gather/evaluate pre- and post- activity pain; help user identify/address circumstances that might impede relaxation and chose strategies to overcome obstacles; discuss benefits and reminders for practicing; plan regular practice times; set and review practice goals.
**3**	**Activity/rest cycling**	Review prior session content and practices; Introduce concept of activity/rest cycling; identify activities user tends to overdo; vicarious learning exercise demonstrate how to change overdone activities; create personal plan to fit daily routine and personal goals; review how other skills help with use of this one; plan regular practice times; set and review practice goals.
**4**	**Pleasant activity scheduling and identify negative automatic thoughts**	Review prior session content and practices; Introduce concept of pleasant activity scheduling; lead user through exercise for adding pleasant activities to their lives; mini-practice of 10-minute pleasant activity to be done immediately (gather/ evaluate pre- and post- activity pain); schedule 3 pleasant activities for week; problem-solve barriers with interactive vicarious learning exercise; Introduce concept of negative automatic thoughts; describe connections between thoughts, emotions, behaviors, and pain; walk user through a thoughts exercise; plan regular practices; set and review practice goals.
**5**	**Identify/change negative automatic thoughts and coping thoughts**	Review prior session content and practices; Continue and advance prior session’s activities related to automatic thoughts and introduce coping thoughts. Practice identifying negative thoughts and accompanying emotional and physical reactions of virtual patients, then self; exercise to teach generation of alternative thoughts, then practice and record accompanying sensations. Focus on teaching generation of alternative thoughts, practice generating calming self-statements; practice skills and get feedback; identify and address circumstances that impede use of these skills and strategies to overcome obstacles; “mini-practices” for specific circumstances; plan regular practices; set and review practice goals.
**6**	**Pleasant imagery and distraction techniques**	Review prior session content and practices; Introduce pleasant imagery and auditory and focal point distraction techniques; complete exercises with audio instructions; plan regular practices; set and review practice goals.
**7**	**Problem Solving**	Review prior session content and practices;; Introduce concept of problem solving and describe steps; demonstrate problem solving with character stories; generate list of challenging situations; exercise to help users select skills for each situation, with personalized plan for overcoming barriers; plan regular practices; set and review practice goals.
**8**	**Monitoring for maintenance**	Review all session content; evaluate skill frequency, helpfulness and comparison to other users; exercises to develop plan for maintenance of skills; motivate further practice and skill development; remind how skills facilitate personal goals; review practice goals.
	**Booster sessions**	Review module 8 as well as revisit any useful/meaningful sessions.

A variety of program features were designed to enhance engagement and adherence [43]. For instance, participants are led through the program by a female virtual “coach” who speaks directly to them as she provides verbal instruction, feedback, and encouragement. Her dialogue is accentuated with changing images (e.g., illustrative graphics or pictures of the coach that change so that her body language and facial features correspond to ideas being expressed) and onscreen text highlighting key ideas. In addition, theoretically-based methods drawn from social cognitive theory [44, 45], adult learning theory [46, 47], and principles of multimedia instruction [e.g., 48] are used to reinforce skill learning and mastery. The overall approach is consistent with research suggesting that certain combinations of text, audio, animation, and graphics can enhance learning e.g., [48].

In addition, PainCOACH includes supplementary features to support use of new skills. For instance, *COACHtrack* promotes self-monitoring by allowing participants to review and change practice goals, record practices and “coping confidence” (self-efficacy), view graphic summaries of their progress over time, and manage automated practice reminders. Another feature, called *COACHchat*, allows them to read about others’ experiences using PCST and to submit descriptions of their own experiences. It was designed to provide benefits of observational learning that people get in face-to-face PCST groups. Finally, *MyCOACH* provides information about the program, the study, and actions to take in a medical or mental health emergency. A companion workbook provides instructions for using PainCOACH, an overview of its modules and features, and worksheets and practice logs.

Finally, participants allocated to the intervention group will receive a *physiotherapist-guided home-based exercise program* primarily designed to strengthen lower limb muscles. It will be progressed over the program to maintain a moderate intensity. Exercises will be prescribed based on our own previous exercise program used for face-to-face interventions in people with knee OA [49]. They have been shown to improve pain and physical function [50] and reflect current clinical practice (Table 2). Participants will be prescribed a minimum of five and a maximum of six exercises. These will include two knee extensor strengthening exercises, one each of a hip abductor, hamstring, and calf strengthening exercise and one additional exercise (from a limited list) chosen by the physiotherapist based on assessment. Participants will be directed to online instructions and video demonstrations of the home exercises to help guide the exercises set by the physiotherapist. They will also be encouraged to increase their overall level of physical activity and will receive written information about ways to do so as well as the option of using a pedometer as a motivational tool.

**Table 2:** **Home exercise program protocol**

Maximum of 6 exercises (with progression as appropriate)			
	2 knee extensor strengthening exercises		
	1 hip abductor strengthening exercise		
	1 hamstring strengthening exercise		
	1 calf strengthening exercise		
	1 other exercise chosen based on assessment findings		
**1. Quads strengthening (each program must include 2 exercises)**			
**Knee extension**	Non weight-bearing	A. Seated knee extension (with resistance) with 5 second hold	**Indications:** suggested as an initial exercise
			**Progression:** Increase cuff weight or theraband resistance – red through to black
			**Simplification:** eliminate weight or see 1B
	Non weight-bearing	B. Inner range quads over roll (with resistance) with 5 second hold	**Indications:** Usually only required when any flair ups with seated knee extension (1A)
			**Progression:** Use appropriate level of ankle cuff weight
			**Simplification:** eliminate weight if flare up
**Sit-to-stand**	Weight-bearing	C. Sit to stand without using hands	**Indications:** suggested as an initial exercise
			**Progression:** lower chair height, hover above the seat without touching down, more weight on affected leg, slit leg position (affected leg closer to seat)
			**Simplification:** use hands
**Steps**	Weight-bearing	D. Step-ups	**Indications:** suitable progression from sit to stand (1C)
			**Progression:** Increase step height, hold extra weight (in hands or backpack)
			**Simplification:** sit to stand (1C)
	Weight-bearing	E. Forward touchdowns from a step	**Indications:** suitable progression from step-ups (1D)
			**Progression:** Increase step height, hold extra weight (in hands or backpack), don’t touch down
			**Simplification:** step-ups (1D)
**Partial squats**	Weight-bearing	F. Partial wall squats	**Indications:** suitable progression from sit to stand (1C)
			**Progression:** Increase to 5 sec hold, more weight on study side)
			**Simplification:** if find flare/problematic step back to sit to stand (1C)
**2. Hip abductor strengthening (1 exercise)**			
**Standing hip abduction**	Non weight-bearing	A. Side leg raises in standing	**Indications:** suggested as an initial exercise
			**Progression**: Increase cuff weight or theraband resistance – red through to black
			**Simplification:** eliminate weight
**Side stepping**	Weight-bearing	B. Crab walk with resistance band	**Indications:** good progression from standing leg side raises (2A)
			**Progression:** Increase theraband resistance – red through to black
			**Simplification:** side leg raises in standing (2A)
**Standing hip abduction**	Weight bearing	C. Wall push standing on study leg	**Indications:** good progression from crab walking (2B) and for variety at final session
			**Progression:** Increase step height. Hold extra weight (in hands or backpack)
			**Simplification:** If unable to tolerate static standing on joint then avoid and use 2B or 2A. Precaution in those with increased varus.
**3. Hamstring strengthening (1 exercise)**			
**Standing knee flexion**	Non weight-bearing	Standing over bench knee curls with weight	**Progression:** Increase cuff weight or theraband resistance – red through to black
			**Simplification:** eliminate weight
**4. Calf strengthening (1 exercise)**			
**Standing plantar-flexion**	Weight-bearing	Double heel raises	**Progression:** single heel raises, raises from the edge of a step
**5. Others (1 exercise if appropriate)**			
**Knee ROM**	Weight bearing	A. Deep squats holding onto a bench/chair	**Progression:** increase squat depth
**Hip ROM**	Weight bearing	B. Deep lunges holding onto back of chair/bench	**Progression:** increase lunge depth
**Hip extensors**	Weight-bearing	C. Bridging	**Progression:** split leg bridge, single bridge with a hold, bridging one leg

Using the Internet to mediate learning, the physiotherapist will begin each participant’s exercise program by selecting exercises and prescribing dosages based on findings from an initial assessment of the participant’s pain and perceived level of effort during performance of an exercise. The participant will learn each exercise by observing as the physiotherapist demonstrates the exercise and then performing it under the physiotherapist’s supervision.

The physiotherapist will perform a brief assessment at each physiotherapy session to ascertain any adverse effects that may have occurred with home exercises and check quality and form of exercise performance. Progression of exercises will be provided by varying the type of exercise as well as the number of repetitions, load or degree of difficulty within an exercise. The prescribed resistance will aim to approximate a 10-repetition maximum level and a patient-rated level of effort experienced during each strengthening exercise of at least 5 out of 10 (hard) on a modified Borg Rating of Perceived Exertion CR-10 scale designed specifically for strengthening exercise [51].

In order to minimise burden of exercise, only the study knee will be the focus of treatment and evaluation. If participants have bilateral symptoms, the physiotherapist may choose exercises that are performed in weight-bearing on both legs simultaneously to achieve bilateral strength gains within the constraints of the treatment protocol.

Although some discomfort is expected during the exercise intervention, any exercise-related pain should subside to usual levels by the next day with no increase in knee swelling following the exercise session. Participants will be taught how to determine whether pain levels are acceptable or not during and for a short time after the exercises. If a specific exercise is aggravating the participant’s pain, then the physiotherapist will reduce the resistance, dosage and/or level of challenge within the exercise until the pain flare settles or change the exercise completely if it remains pain provoking.

#### Treatment fidelity

Eight experienced musculoskeletal physiotherapists (4 female, 4 male) in 7 locations around metropolitan Melbourne and regional Victoria have been selected and trained to deliver the study exercise intervention. They have an average of 16 years (minimum 3 years, maximum 28 years) of clinical experience treating musculoskeletal disorders. They attended a full-day training course in Melbourne conducted by the researchers on February 22nd 2014 and were provided with a detailed intervention manual. Training included information about study procedures and protocol, exercise delivery, progression and monitoring. As therapists are encouraged to support and reinforce pain coping skills taught on PainCOACH, they also received background information, exposure and education about those skills so they can be integrated into the exercise program and daily life. Regular telephone/Skype meetings between the physiotherapists and a member of the research team will be held throughout the study. Physiotherapists will complete standardised treatment notes for each participant’s individual treatment session and return these via an online survey within 48 hours of the session appointment. Treatment fidelity will be assessed using a treatment check list applied to key points within the treatment notes.

### Descriptive data

Age, gender, duration of knee symptoms, previous treatments, medical history, and medication use will be obtained at baseline by an online questionnaire.

### Outcome measures

The outcome measures are summarised in Table 3. Primary outcomes will be measured at baseline, 3 months and 9 months. Conclusions regarding treatment effectiveness will be evaluated based on changes in primary outcome measures from baseline to 3 months. Our two primary outcomes, which are recommended as valid measures of pain and physical function for knee OA [52], are:

**Table 3:** **Summary of outcome measures and time points**

Primary outcome measures	Data collection instrument	Collection points
Average walking pain in past week	11-point numeric rating scale	0, 3^¥^, and 9 months
Physical function in past 48 hours	WOMAC osteoarthritis Index physical function subscale	0, 3^¥^, and 9 months
**Secondary outcome measures**		
Pain in past 48 hours	WOMAC osteoarthritis Index pain subscale	0, 3 and 9 months
Perceived change overall	7-point ordinal scale	3 and 9 months
Perceived change in pain	7-point ordinal scale	3 and 9 months
Perceived change in function	7-point ordinal scale	3 and 9 months
Health-related quality of life	Assessment of Quality of Life questionnaire (AQoL2)	0, 3 and 9 months
Self-reported psychological measures	Arthritis self-efficacy scale	0, 3 and 9 months
	Coping Strategies questionnaire (CSQ)	0, 3 and 9 months
	Pain Catastrophizing Scale (PCS)	0, 3 and 9 months
**Other measures**		
Adherence to intervention	Number of physiotherapy sessions attended	During intervention
	Weekly home exercise log books	During intervention
	PainCOACH module completion	During intervention
	PainCOACH practice via COACHTrack/ log books	During intervention
	Home exercises - 11-point numeric rating scale	3, 6 and 9 months
	Frequency of home exercise sessions previous 2 weeks	3, 6 and 9 months
	Frequency of pain coping skills in previous 2 weeks	3, 6 and 9 months
	Educational material accessed in previous 3 months	3, 6 and 9 months
Adverse events and harms	Questionnaire	3, 6 and 9 months
Health cost data	Questionnaire	9 months
Descriptive information	Questionnaire	0 months
Medications and co-interventions	Questionnaire	0, 3, 9 months
Treatment benefit expectations	5-point ordinal scale	0 months
**Process measures**		
Treatment satisfaction	Questionnaire	3 months
Appropriateness of the intervention	Program (System) usability scale	3 months

^¥^Primary outcomes and primary time point.

#### Knee pain on walking measured by an 11-point NRS

Average knee pain on walking over the past week will be self-reported using an 11-point numeric rating scale (NRS) (0 = no pain, 10 = worst pain possible). This measurement is reliable and valid in OA populations [53]. The minimum clinically important difference to be detected in OA trials has been defined as a change in pain of 1.8 units (out of 10) [54].

#### Physical function measured by the function subscale of the WOMAC

Physical function will be measured by the WOMAC Osteoarthritis Index (Likert version 3.1) [55]. This is a disease-specific self-report questionnaire with extensive evidence of validity, reliability and responsiveness in OA populations [56]. The physical function subscale contains 17 questions (each answered on a Likert scale where 0 = no dysfunction and 4 = extreme dysfunction) and has a total score ranging from 0 (no dysfunction) to 68 (maximum dysfunction). The minimum clinically important difference to be detected in OA trials has been defined as a change in function of 6 points (out of 68) [57].

Our secondary outcome measures, which will collected at baseline, 3 and 9 months, unless otherwise indicated below, include:

#### Participant-perceived response to treatment measured on 7-point scales

Participants will rate their perceived change in a) pain, b) physical function and c) overall condition on a seven-point ordinal scale (1-much worse to 7-much better) at 3 and 9 months. This scale will be used as an external criterion for comparison with changes in scores of other outcomes [58]. Measuring participant-perceived change using a rating of change scale has been shown to be a reliable and clinically relevant method of identifying improvements that are truly meaningful from the individual perspective [59]. Participants who report that they are moderately better and above will be classified as improved.

#### Knee pain measured by the WOMAC pain subscale

Knee pain will also be captured using the WOMAC pain subscale, which contains five questions, each answered on a Likert scale where 0 = no pain and 4 = extreme pain. It has a total score ranging from 0 (no pain) to 20 (maximum pain) [55].

#### Health-related quality of life measured by the Assessment of Quality of Life (AQoL)

The AQoL questionnaire (version AQoL-II) contains 20 questions that cover six dimensions of health-related quality of life including independent living, social relationships, physical senses, coping, pain and psychological wellbeing. Responses to each question are answered on 5-point scale. Scores range from -0.04 (worst possible health-related quality of life) to 1.00 (full health-related quality of life). The AQoL-II has strong evidence of validity and responsiveness [60, 61]. A clinically important difference in health-related quality of life has been defined as a change of 0.04 AQoL units [62].

#### Self-efficacy for pain and function measured by the arthritis self-efficacy scale

Self-efficacy for pain management and its effects on function will be measured with the Arthritis Self-Efficacy Scale [63], which assess confidence for managing pain (5 questions) and physical function (9 questions) on a 10-point NRS (where 1 = very uncertain and 10 = very certain). Responses are averaged so that higher scores indicate greater self-efficacy. This scale is reliable and valid in OA populations [63].

#### Pain catastrophising using the pain catastrophising scale

The Pain Catastrophising Scale measures the tendency to ruminate about pain, magnify pain, and feel helpless about pain. All 13 items are measured on a 5-point Likert scale (where 0 = low levels of catastrophising and 4 = high levels of catastrophising). The highest possible total score of 52 indicates the greatest level of catastrophising. It has high internal consistency and is associated with heightened pain, psychological distress, and physical disability [64].

#### Use of coping skills to manage pain using the coping attempts scale of the Coping Strategies Questionnaire (CSQ)

The CSQ [65] can be used to measure how often a patient uses six cognitive and behavioural pain coping strategies to manage pain (diverting attention, reinterpreting pain sensations, coping self-statements, ignoring pain sensations, praying and hoping, and increasing activity level). Items are measured on a 7-point Likert scale (where 0 = never uses coping skills and 7 = always uses coping skills). Based on prior factor analyses of this instrument [66], participant’s responses will be converted to scores on the Coping Attempts factor of the CSQ. The CSQ has demonstrated sensitivity to change from treatment in chronic pain samples as well as good internal consistency and construct validity [66].

### Other measures

#### Treatment adherence

The number of Skype physiotherapy sessions attended by each participant will be recorded by the treating physiotherapist and submitted to the researchers using scheduled online treatment notes. The number of PainCOACH sessions completed will be automatically recorded by the program progress log which will be monitored regularly by the researchers. During the intervention period, adherence to the home exercise program will be self-reported in weekly log books, which at the completion of the intervention will either be transcribed to an online survey or returned by post, depending on participant preference. Pain coping skills practice and coping confidence (self-efficacy for using pain coping skills to manage pain) will be recorded using the PainCOACH practice tracking log (COACHtrack) or alternatively by completing weekly logs that will either be transcribed to an online survey or returned by post.

Participants in the PCST + Ex group will also rate their adherence to their home exercise program over the previous 3 months (from ‘not at all’ to ‘completely as instructed’) at 3, 6 and 9 months using an online 11-point NRS. They will also report the number of exercise sessions they completed and how often they used the PainCOACH skills in the previous 2 weeks, at 3, 6, and 9 months. Participants in both groups will report which online education information sheets were accessed in the past 3 months 3, 6, and 9 months using an online checklist.

#### Adverse events and use of health services/co-interventions

Adverse events will be defined as any health problems that participants: i) believed to be caused by the treatment; ii) that required them to see a health professional or take medications; iii) and/or that interfered with function for 2 or more days. Information on adverse events throughout the 13 week intervention will be collected for the combined group via exercise log books and at 3, 6 and 9 months for both groups via an online survey. Participants’ use of health services and co-interventions (medication for knee pain and any other treatment for knee pain) will be collected via an online survey at 0, 3 and 9 months. Direct health care costs and direct non-health care resources will be collected at the 9 months via an online survey.

#### Expectations of treatment effect

At baseline, participants will be asked about their expectations of the effect they think the study treatments will have on their knee pain using a 5-point ordinal scale ranging from no effect to complete recovery.

#### Process measures

Additional process measures will be collected for both groups at 3 months for the purposes of measuring the acceptability of the interventions (rather than to determine treatment efficacy). These additional measures include: i) *appropriateness of the interventions* using a custom designed questionnaire and ii) *satisfaction with the intervention* using a 2-item custom designed questionnaire that asks about the overall satisfaction with the content and delivery of the intervention.

### Sample size

The primary endpoints of the trial are between-group (PCST + Ex group versus the educational control group) differences in change in knee pain on walking (NRS) and change in physical function (WOMAC). Moderate between-group treatment effects of around 0.5 have previously been reported for pain and function following individual exercise programs in people with knee OA [13, 67]. Therefore, to enable detection of at least a 0.5 effect size between groups in either pain or function in a two-arm trial with 80% power, a significance level at 0.05 and allowing for 15% attrition rate, we will recruit 74 participants in each group or a total of 148 participants.

### Data and statistical analysis

A biostatistician (MPS) blinded to group allocation will analyse data. Main comparative analyses between groups will be performed using intention-to-treat. This analysis will include all participants who have missing data and those who do not fully adhere to the protocol. Demographic characteristics, as well as baseline scores on primary and secondary outcome measures, will be inspected to assess baseline comparability of treatment groups. These variables will also be examined for those participants who withdraw from the study and those who remain. Descriptive statistics will be presented for each group as the mean change (standard deviation, 95% confidence intervals) in the outcomes from baseline to each time-point. For continuous outcomes differences in mean change will be compared between groups using linear regression random effects modelling adjusted for baseline values of the outcome. Proportional odds models will compare improvement between groups based on perceived ratings of change as well as the proportion in each group who attain the minimum clinical important difference (MCID) for pain and function. The MCID to be detected in OA trials is a change in pain of 1.8 units (out of 10) [54] and change in function of 6 points (out of 68) [57]. Model diagnostic checks will utilise residual plots. Similar regression models for binary and ordinal outcome measures will use random effects logistic and proportional odds models, respectively. We will also perform a per protocol analysis as appropriate.

### Timelines

Ethics approval was obtained from the Human Research Ethics Committee of the University of Melbourne in August 2013. Recruitment and training of the physiotherapists was completed during February 2014. The trial was registered with the Australian and Zealand Clinical Trials Registry reference: ACTRN12614000243617 in March 2014 prior to participant recruitment which commenced in March 2014 and is expected to be completed by June 2015. The trial is due for completion in March 2016 when all participants are expected to have completed the 9-month follow up.

## Discussion

This paper provides the justification and the protocol for a pragmatic RCT that will investigate whether Internet-based PCST + Ex is more effective than online educational material alone in people with persistent knee pain consistent with a clinical diagnosis of knee OA. The findings of this study will help determine if a PCST and exercise intervention that is delivered using the Internet over 3 months can improve self-reported pain and physical function when compared to an educational control.

Identification of feasible healthcare models that improve outcomes in people with persistent knee pain and knee OA has important implications for clinical practice. Our study will provide new information about the effectiveness and acceptability of a novel clinical practice model for the delivery of interventions that are known to be beneficial to people with persistent knee pain. Findings could therefore have the potential to guide clinical practice towards innovative modes of healthcare provision. This does not diminish the importance of usual methods of delivery of health care, such as face-to-face visits when access is available, however this model could be of particular benefit to people in regional and remote areas who may not be able to access face-to-face care.

In order to maximise translatability, this study was intentionally designed to test a service delivery model that could be easily implemented. Use of a limited number of physiotherapy contacts similar to usual care, a home-based exercise program, and the Internet as a vehicle to deliver the intervention, which is becoming more widely accessible and acceptable to this patient population, will assist this endeavour. If the results of our proposed study support the benefits of Internet-based PCST + Ex, the components of this intervention could be easily implemented into clinical practice.

Strengths of this RCT study design are the pragmatic nature of treatment delivery by practising physiotherapists as well as the reproducibility of both the physiotherapy and pain coping skills programs. These features will improve the ability to translate the findings into a range of health care settings and enable future researchers to replicate the intervention. Importantly, the physiotherapy-guided home exercise program is individualised with regard to the content, and the intensity level of the exercises are individually monitored, progressed or stepped back throughout the intervention according to participant feedback. Additionally, physiotherapists will support and reinforce the participants’ use of the pain coping skills during the exercise sessions so as these skills can not only be integrated into the exercise program, but also encouraged within daily life. The inclusion of participants regardless of their geographic location will further enhance the generalisability of results. The study is adequately powered for our primary outcome measures.

## Authors’ original submitted files for images

Below are the links to the authors’ original submitted files for images. Authors’ original file for figure 1

## Abbreviations

AQoL: Assessment of quality of life
CONSORT: Consolidated standards of reporting trials
CSQ: Coping strategies questionnaire
MCID: Minimal clinical important difference
NRS: Numeric rating scale
OA: Osteoarthritis
PCST: Pain coping skills training
RCT: Randomised controlled trial
WOMAC: Western Ontario McMaster Universities osteoarthritis index.
