# Supplementary figures and images for: Internet-mediated physiotherapy and pain coping skills training for people with persistent knee pain (IMPACT – knee pain): a randomised controlled trial protocol
Source: BMC Musculoskelet Disord. 2014 Aug 13;15:279. doi: 10.1186/1471-2474-15-279 (PMC4137067; doi:10.1186/1471-2474-15-279)

## Enrolment

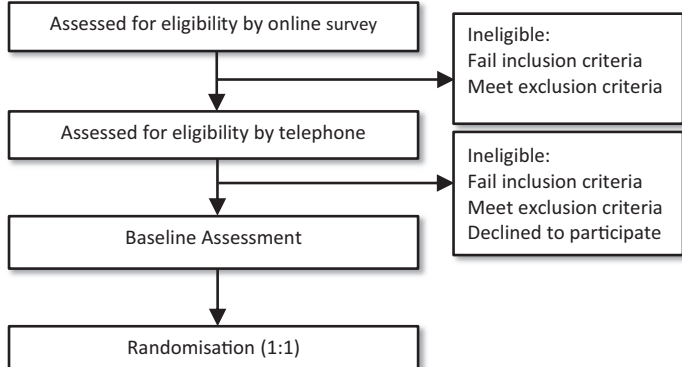

## Allocation

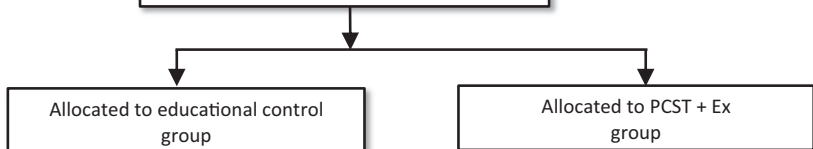Intervention  
(3 months)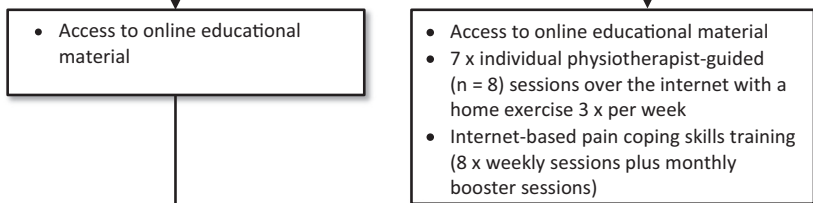

## Follow up

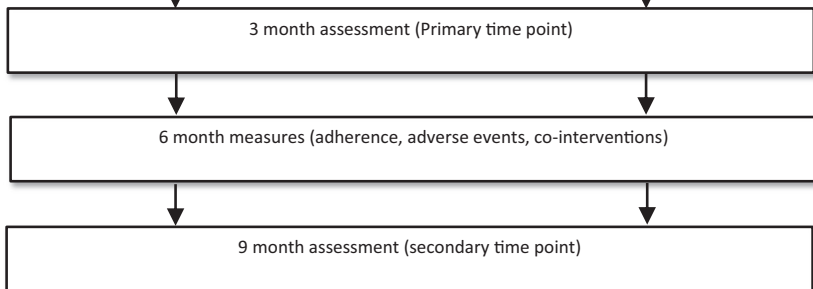

## Analysis

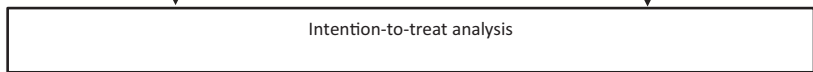

Supplement: Supplementary file 1 — Authors’ original file for figure 1 [file 12891_2014_2219_MOESM1_ESM.pdf]
